# Gender difference analysis of Xp11.2 translocation renal cell carcinomas’s attack rate: a meta-analysis and systematic review

**DOI:** 10.1186/s12894-020-00696-1

**Published:** 2020-08-26

**Authors:** Wenyuan Zhuang, Ning Liu, Hongqian Guo, Chunni Zhang, Weidong Gan

**Affiliations:** 1 Department of Urology, Nanjing Drum Tower Hospital, Medical School of Nanjing University, Institute of Urology, Nanjing University, 321 Zhongshan Road, Nanjing, 210008 China; 2 Department of Clinical Laboratory, Jinling Hospital, State Key Laboratory of Analytical Chemistry for Life Science, Jiangsu Engineering Research Center for MicroRNA Biology and Biotechnology, Nanjing University School of Medicine, Nanjing University, Nanjing, China

**Keywords:** Xp11.2, tRCC, TFE3, Gender, X chromosome

## Abstract

**Background:**

Xp11.2 translocation renal cell carcinoma (tRCC) is recently recognized. As Xp11.2 tRCC involved gene translocation and fusion in X chromosome and the number of X chromosomes in female is twice of male, we wondered whether the gender difference of attack rate is consistent with the proportion of the X chromosome. ***Methods*:** In the present paper, meta-analysis was performed to find out the difference of morbidity between male and female.

**Results:**

Nine studies with 209 cases calculated. Odds ratios (ORs) and ORs with 95% confidence intervals (CIs) were calculated for attack rate of Xp11.2 RCC with different gender. The result showed that the attack rate of female was higher than that of male with pooled OR of 2.84 (95% CI = 1.48–5.45), while the rate rises even further in adult (OR = 3.37, 95% CI =2.19–5.18). In other types of common kidney cancer, the OR value is less than 1, which means that the incidence of female is lower than that of male.

**Conclusions:**

The result showed that the incidence rate of female patients is much higher than that of male patients with Xp11.2 tRCC, it was reasonable to indicate that this particular incidence rate is related to the X chromosome.

## Background

Xp11.2 translocation renal cell carcinoma (tRCC) was delineated as a distinct entity in the 2004 World Health Organization (WHO) renal tumor classification [1]. Recently, the published WHO classification of tumors classified the Xp11.2 tRCC as one of MiT (microphthalmia transcription factor) family tRCC [2]. A total of 5 Xp11.2 tRCCs have been identified in RCC tumors, *PRCC-TFE3, ASPSCR1-TFE3, SFPQ-TFE3, NONO-TFE3,* and *CLTC-TFE3,* all of which result in TFE3 (transcription factor binding to IGHM enhancer 3) gene fusions [3]. TFE3 gene is located on the short arm of the X chromosome (Xp11.2). The functional domain of the TFE3 gene fused with the promoter of other genes, housekeeping gene usually, resulting to the TFE3 protein is constitutively overexpressed in Xp11.2 tRCC which can be specifically identified by IHC (immunohistochemistry) [4, 5].

Xp11.2 tRCC is predominantly reported in children and young adults less than 45 years of age with a one-third incidence in juveniles [6]. Adult patients are rare reported with an incident rate of 1%. Xp11.2 tRCC shared similar feature to conventional clear cell and papillary renal carcinomas in histology, which creates diagnostic difficulties, and the fact is one possibility to explain the problem of its frequency [7, 8]. However, Xp11.2 tRCC showed more aggressive behavior, with metastasis common at presentation, and poorer prognosis than other subtypes of RCC. In addition, previous studies revealed that Xp11.2 tRCC was inherently more aggressive in adults than that in children [9]. Complete surgical removal of the tumor mass including the kidney may be the preferred therapy in patients with lower stage tumors. Today, the clinical characteristic and epidemiology of Xp11.2 tRCC are not very clear for the rarity of Xp11.2 tRCC. Controversy about the gender difference of morbidity remained unclear. Several published studies showed the female predominance in incidence of Xp11.2 tRCC while a few studies reported it is seen more often in males than in females [1].

As Xp11.2 tRCC involved gene translocation and fusion in X chromosome and the number of X chromosomes in female is twice of male, we are interested in the relation of female predominance in attack rate of Xp11.2 tRCC and the sex chromosome. We wondered whether the gender difference of attack rate is consistent with the proportion of the X chromosome. Gender factor was significantly associated with the frequency of many diseases and Xp11.2 tRCC might be one of them [10, 11]. However, up to date, studies on large sample for analysis of the clinical characteristic and epidemiology of Xp11.2 tRCC were still lack. Therefore, we analyzed the difference of morbidity between male and female in this systematic review by using meta-analysis and investigated the potential role of X chromosome on Xp11.2 tRCC. For comparison, we also analyzed clear cell carcinoma (ccRCC), papillary cell carcinoma (pRCC), and chromophobe cell carcinoma (ChRCC).

## Methods

### Literature search and study selection

**Fig. 1:**
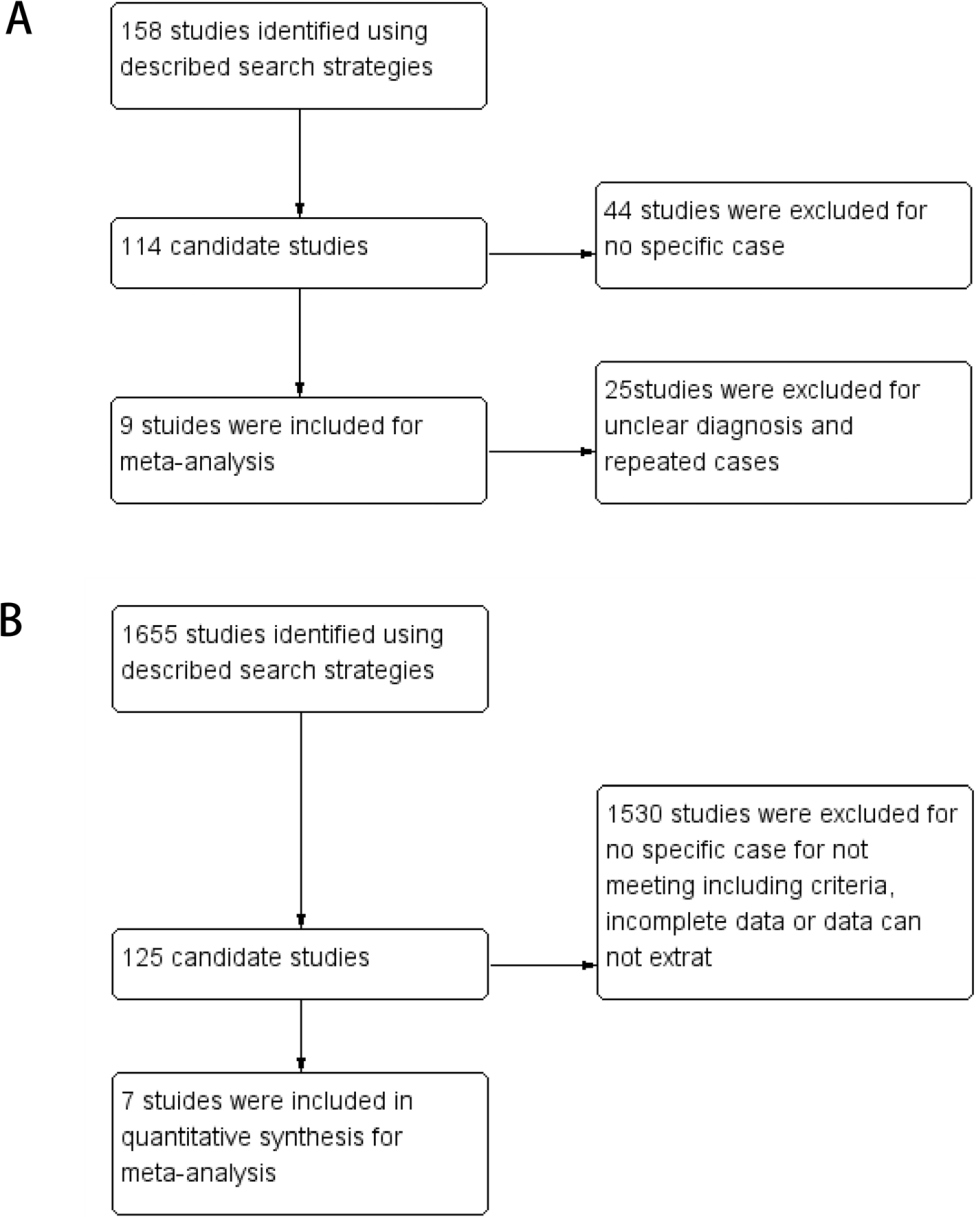
Flow diagram of study selection. **a** Studies of Xp11.2 tRCC; **b** Studies of ccRCC, pRCC and ChRCC

A computer-aided literature search was performed with usage of the Cochrane, DARE, MEDLINE, EMBASE, and Science Citation Index. The literature published between July 2004 and May 2019 was searched. An initial search strategy used recognized search terms “TFE3” and “Xp11.2 renal cell carcinoma”. Primary studies that meet following criteria were included: (1) All studies that reported cases with definite gender; (2) All cases were confirmed by specific biological technology, such as IHC assay for TFE3, FISH, RT-PCR, and/or other molecular biology methods. (3) The report contained enough cases (≥10). Reviews and mechanism researches were excluded. Studies that performed in the lab with animal or cell models were also excluded. We also collected common types of renal cell carcinoma for comparison, searches included the terms “clear cell renal cell carcinoma” and “papillary renal cell carcinoma” and “chromophobe renal carcinoma”. The citations listed in the retrieved articles were reviewed to identify other potentially relevant studies. Primary studies that meet following criteria were included: (1) The patient was diagnosed as renal cell carcinoma. (2) Patients have clear data of treatment and follow-up. (3) There is sufficient data for analysis. All studies were evaluated carefully to eliminate duplicate patient populations. Figure 1 shows a flow diagram.

### Data synthesis and analysis

We paid our attention to the difference of morbidity between male and female and all primary studies with gender data were analyzed. The statistical software Review Manager (Version 5.3 for Windows) was applied to carry out all the analysis. Dichotomous data which incidence regarded were expressed as odds ratios (ORs) and ORs with 95% confidence intervals (CIs) were used in the Mantel-Haenszel fixed-effect model when no statistically significant heterogeneity was detected. On the contrary, Mantel-Haenszel random-effect model would be chosen when heterogeneity was significant. Heterogeneity analysis was performed using the Cochran *Q*-test and I^2^ index, the existence of heterogeneity statistically was considered when the *p* value was less than 0.1. On the other hand, evaluation of publication bias was performed for each of the pooled study groups using a funnel plot.

## Results

### Literature and study characteristics

**Fig. 2:**
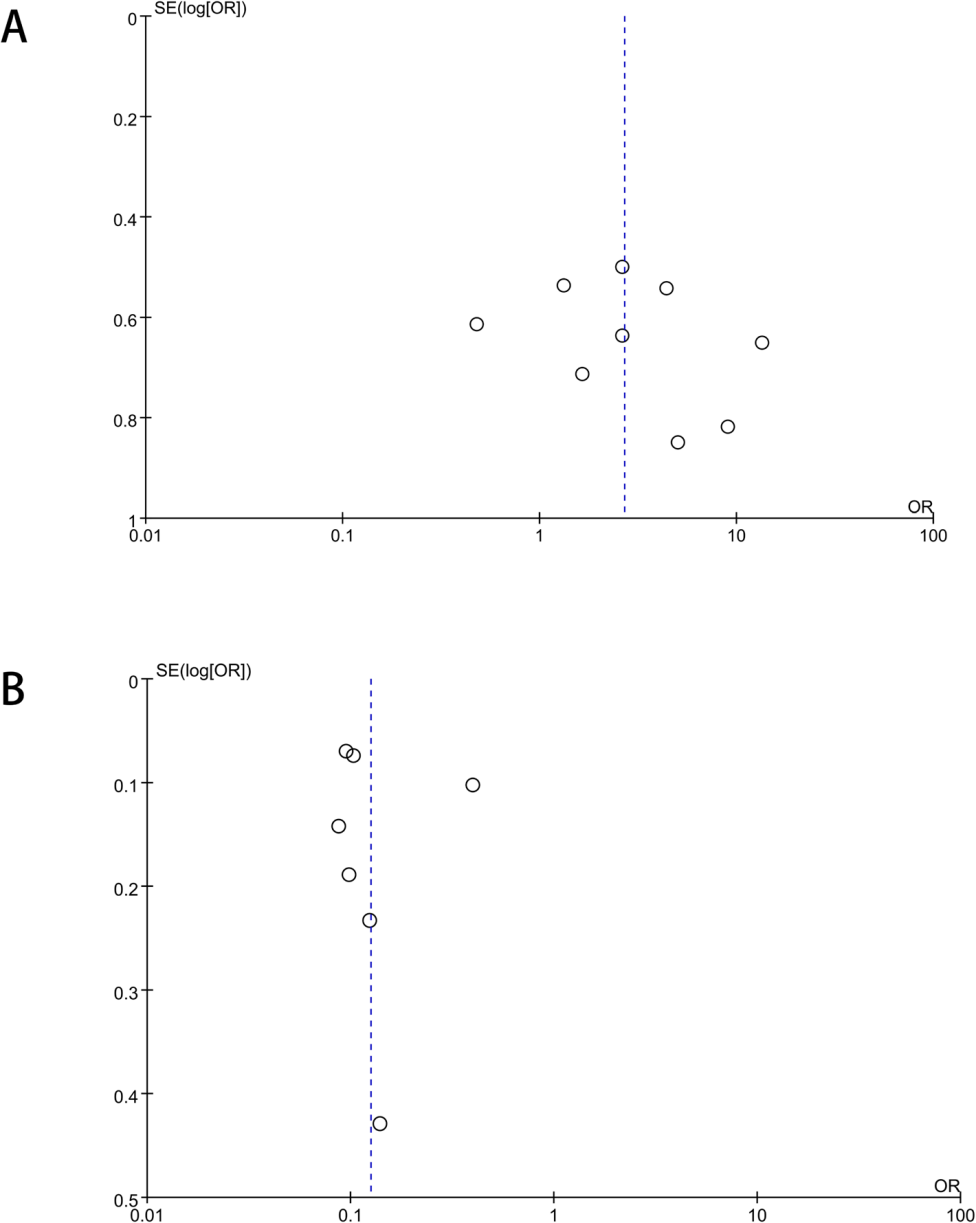
Publication bias assessment for study. Funnel plots show that there was no evidence for significant publication bias in any of the 2 pooled groups. **a** Studies of Xp11.2 tRCC; **b** Studies of ccRCC, pRCC and ChRCC

All published cases of Xp11.2 tRCC were enrolled except a few of cases could not meet the inclusion criteria. Nine studies with 209 cases were collected. It was also checked that all cases were unique, no repeated cases were included. For common types of kidney cancer, 7 studies were included in quantitative synthesis for meta-analysis, as shown in Table 1 and Table 2. Publication bias is described as visual assessment of a funnel plot in Fig. 2 and there was no evidence for significant publication bias.

**Table 1:** Clinical characteristics of included studies with Xp11.2 tRCC

	Cases				LN metastasis				Distant metastases				Mean age	Main diagnostic method	
	male/C	female/C	male/A	female/A	male/C	female/C	male/A	female/A	male/C	female/C	male/A	female/A			
Levevte et al. [12]	1	1	12	14	0/1	0/1	3/12	2/14	0/1	0/1	5/12	6/14	–	FISH	
Chenchen et al. [13]	0	0	7	9	–	–	–	–	–	–	–	–	–	IHC + FISH	
Ning Liu et al. [14]	2	2	11	19	0/2	0/2	1/11	4/19	0/2	0/2	1/11	2/19	44.5 ± 18.3	FISH	
Argani et al. [15]	0	0	6	22	0/0	0/0	2/6	9/22	0/0	0/0	2/6	2/22	37 ± 16.6	IHC + FISH	
Green et al. [16]	0	2	10	19	0/0	2/2	0/10	4/19	0/0	1/2	1/10	2/19	30.2 ± 14.1	IHC + FISH	
Lim et al. [6]	0	1	8	12	0/0	1/1	3/8	2/12	0/0	1/1	0/8	2/12	43.4 ± 20.0	FISH	
Plfueger et al. [17]	3	2	5	12	1/3	0/2	0/5	3/12	1/3	0/2	1/5	0/12	24.6 ± 20.3	IHC + FISH	
Song et al. [18]	13	9	0	0	–	–	–	–	–	–	–	–	–	IHC + FISH	
Pan et al. [19]	1	0	3	9	0/1	0/0	0/3	4/9	0/1	0/0	0/3	4/9	24.9 ± 9.2	IHC + FISH	
total	20	17	72	118	1/7	3/8	9/55	25/97	1/7	2/8	10/55	18/97

**Table 2:** Clinical characteristics of included studies with ccRCC, pRCC and ChRCC

PRCC					
	male	female	LN metastasis	Distant metastases	Mean age
Beck et al. [20]	117	40	–	9/157	62.2 ± 12
Toloken et al. [21]	236	74	7/310	unknown	64
Keengan et al. [22]	1740	538	67/2278	98/2278	–
Sterffens et al. [23]	436	129	50/565	54/565	62.1 ± 11.6
Lee et al. [24]	142	50	unknown	unknown	56.4 ± 13.5
Simone et al. [25]	40	15	13/55	5/55	59.1 ± 14.8
Wagener et al. [26]	1469	474	143/1943	151/1943	–
CCRCC					
	male	female	LN metastasis	Distant metastases	Mean age
Beck et al	487	307	–	95/794	61.5 ± 12
Toloken et al	836	497	23	–	62
Keengan et al	8501	5340	172/13841	1219/12841	–
Sterffens et al	2772	1604	319/4376	618/4376	62.4 ± 11.2
Lee et al	1785	703	–	–	56.1 ± 12.4
Simone et al	599	321	29/920	68/920	59.8 ± 12.6
Wagener et al	3357	2243	210/5600	540/5600	–
Chromophobe					
	male	female	LN metastasis	Distant metastases	Mean age
Beck et al	59	47	–	6/106	57.5 ± 12
Toloken et al	120	100	4	–	59
Keengan et al	568	381	11/949	36/949	–

### Results of the search

#### All collected studies

Totally, there were 131 females and 78 males while all the cases according our inclusion criteria were enrolled in. Previous literature has reported significant gender differences in Xp11.2 tRCC between adults and children, so patients of Xp11.2 tRCC was divided into children (≤ 14 years) and adults (> 14 years) for further analysis. The three most common types of renal cell carcinoma, clear cell carcinoma, papillary cell carcinoma, and chromophobe cell carcinoma, were used for comparative analysis.

#### Gender-related incidence of all included studies of Xp11.2 tRCC

**Fig. 3:**
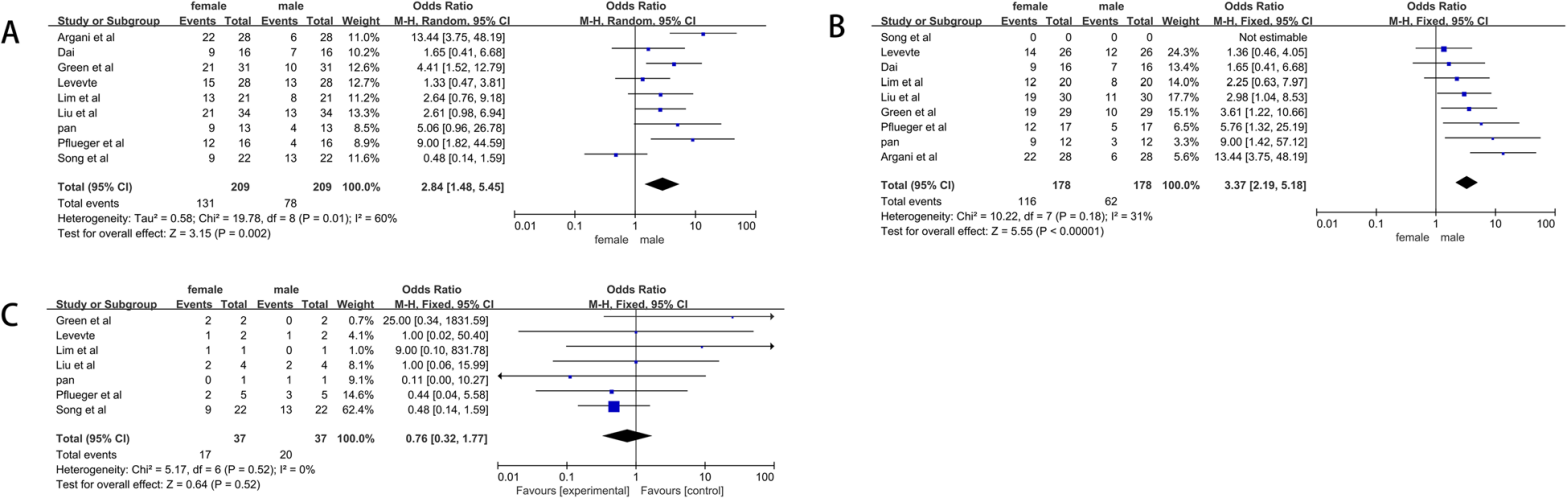
Forrest plots and meta-analysis of studies with different gender-related incidence showing 95% confidence interval in Xp11.2 tRCC; **a** Different gender-related incidence in included studies; **b** Different gender-related incidence in adults; **c** Different gender-related incidence in children

A total of 209 cases with 131 women and 78 men were included. Tests for heterogeneity showed that *P* = 0.002 and I^2^ = 60%. We applied random- effect model while I^2^ value> 50%. Pooled OR was 2.84 (95% CI = 1.48–5.45) (Fig. 3a).

#### Gender-related incidence of included adult studies of Xp11.2 tRCC

A total of 178 cases of Xp11.2 with 116 women and 62 men were included. (*I*^2^ statistic = 31%, *P <* 0.00001), so that Mantel-Haenszel fixed-effect model was still applied. Pooled OR was 3.37 (95% CI =2.19–45.18) (Fig. 3b).

#### Gender-related incidence of included children studies of Xp11.2 tRCC

For this group, there was no evidence for heterogeneity about incidence of different gender (*I*^2^ statistic = 0%, *P =* 0.52). Results of meta-analysis demonstrated that The incidence is similar between male and female with pooled OR of 0.76(95% CI = 0.32–1.77) (Fig. 3c).

#### Gender-related incidence of PRCC, CCRCC and ChRCC

**Fig. 4:**
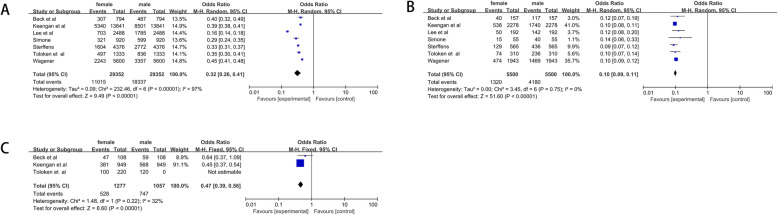
Forrest plots and meta-analysis of studies with different gender-related incidence showing 95% confidence interval in ccRCC, pRCC and ChRCC. **a** Different gender-related incidence in ccRCC; **b** Different gender-related incidence in pRCC; **c** Different gender-related incidence in ChRCC

Seven studies were included in quantitative synthesis for meta-analysis. For pRCC, a total of 5500 cases with 1320 women and 4180 men were included. The pooled OR was 0.10(95% CI = 0.09–0.11) (Fig. 4b). For ccRCC, we got similar results. The pooled OR was 0.32 (95% CI = 0.26–0.41) (Fig. 4a). Results of ChRCC showed that the OR of ChRCC was 0.47(95% CI = 0.39–0.56) (Fig. 4c).

#### Gender-related distant and lymphatic metastases of Xp11.2 tRCC

**Fig. 5:**
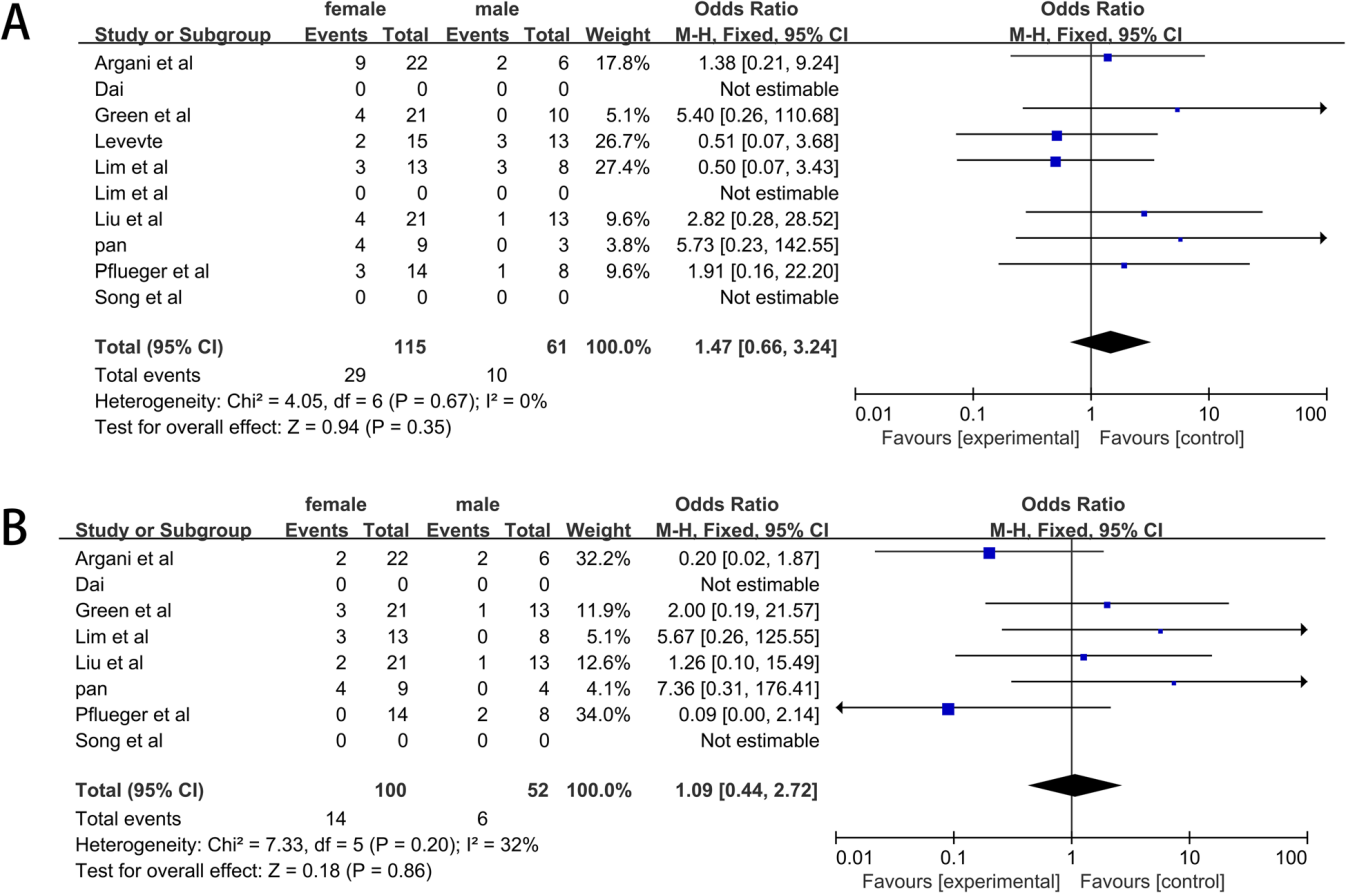
Forrest plots and meta-analysis of studies showing 95% confidence interval of male Xp11.2 tRCC as compared with female Xp11.2 tRCC. **a** Incidence of lymphatic metastases; **b** Incidence of distant metastases

A total of 209 cases with 131 women and 78 men were included. Tests for heterogeneity showed that no evidence of heterogeneity in the incidence of distant metastasis (*I*^2^ statistic = 32%, *P =* 0.86) and lymphatic metastasis (*I*^2^ statistic = 0%, *P =* 0.35) was observed in two pool. The results showed that the rates of lymphatic metastasis (OR = 1.47, 95% CI = 0.66–3.24) (Fig. 5a) and distant metastasis (OR = 1.09, 95% CI = 0.44–1.72) (Fig. 5b) were comparable between male and female patients.

## Discussion

Xp11.2 tRCCs, as rare tumors, seen more often in children and young adults, account for less than 5% of all sporadic kidney cancers. It is reported that the prognosis for Xp11 tRCC is similar to that for clear cell RCC in adult while children with Xp11 tRCC may have a more favorable outcome [12, 13, 27, 28]. Since the first described fusion of TFE3 on the short arm of the X chromosome to chromosome 1q21.2 [*PRCC-TFE3* t(X;1)(p11.2;q21)], several other TFE3 translocation partners have been identified, including at least five different fusion partners of the Xp11.2 chromosome: *ASPL-TFE3, PRCC-TFE3, PSF-TFE3, CLTC-TFE3,* and *NoNo -TFE3* [14–16, 29–31]. Other novel fusion partners in single case, *PARP14, KHSRP,* and *DVL2*, were recently described. Yet despite nearly two decades since the discovery of Xp11.2 tRCCs, the morphology, biological behavior, and molecular biology underlying these cancers remains largely uncharacterized and effective targeted therapies are yet to be identified.

The translocations on chromosome Xp11.2 result in fusions between TFE3 and its respective fusion partners varies and can produce fusions containing differing number of exons in the case of TFE3 and its gene partners. All TFE3 fusion partners have constitutively active gene promoters, so TFE3 fusion proteins are expressed at dramatically higher levels than wild-type TFE3. IHC technology is used commonly to diagnose Xp11.2 tRCC and TFE3 protein is the most distinctive immunohistochemical feature of Xp11 tRCC. This immunologic marker of Xp11.2 tRCC has a relatively high sensitivity and specificity [17, 32]. However, TFE3 IHC can show false-positive results, TFE3 IHC combined with other diagnosis tools, such as break-part TFE3 fluorescence in situ hybridization (FISH), Reverse Transcription-Polymerase Chain Reaction (RT-PCR), and cytogenetic analysis can be effective means to diagnose the Xp11.2 tRCC.

Xp11.2 tRCC showed significant differences between men and women compared with the common renal cell carcinoma. According to our results, the incidence of Xp11.2 tRCC is much higher in women than in men (OR = 2.84, 95% CI = 1.48–5.45). In adults, the rate rises even further (OR = 3.37, 95% CI =2.19–5.18). Among the three most common types of kidney cancer, women had a lower incidence than men. We suggest that this significant difference may be due to the particular pathogenesis of Xp11.2 tRCC. In order to balance the difference of X chromosomes between male and female, female have two different X chromosomes, one of them is active (Xa) and the other one is inactive (Xi) while male only have one Xa chromosome. An lncRNA, the X-inactive specific transcript (Xist), is selectively expressed and physically coats one of the X chromosomes in the female, resulting in the one of X chromosomes inactivation [18, 19, 33, 34]. The cause of Xp11.2 tRCC is the fusion of TFE3 on the short arm of the X chromosome. There are differences in the number of X chromosomes between men and women and X chromosome inactivation exists in women. We wonder if there was any effect on the different incidence between men and women. Whether this difference in incidence between men and women is related to the number of X chromosomes, and what role Xa and Xi play respectively, these questions are worthy of further study. The incidence of Xp11.2 tRCC varies between adults and children. Gender differences in child morbidity were not compared due to the small number of children in the included sample. However, in terms of the number of cases alone, the number of male and female cases in children appears to be equal. In other respects, there seems to be no significant difference between men and women in terms of lymphatic metastasis (OR = 1.47, 95% CI = 0.66–3.24) and distant metastasis (OR = 1.09, 95% CI = 0.44–1.72).

## Conclusions

Therefore, compared to men, women have a higher incidence for patients with Xp11.2 tRCC. The outcome came out to the difference of X chromosomes between male and female. On the other hand, for the existence of three kinds X chromosomes, Xa of female, Xi of female, and Xa of male, the diversity of the X chromosome may lead to the different source of TFE3 fusion gene. Therefore, it could speculate that the distinction had something to do with the clinical stages, distant metastasis, and prognosis. Further work should be done to confirm this conjecture.

## Data Availability

The data that support the findings of this study are available on request from the corresponding author Weidong Gan. The data are not publicly available due to them containing information that could compromise research participant privacy.

## Abbreviations

RCC: Renal cell carcinoma
ccRCC: Clear cell renal cell carcinoma
pRCC: Papillary renal cell carcinoma
ChRCC: Chromophobe renal cell carcinoma
IHC: Immunohistochemistry
FISH: Fluorescence in situ hybridization
TFE3: Transcription factor binding to IGHM enhancer 3
RT-PCR: Transcription-polymerase chain reaction
